# Prognostic and clinical impact of PD-L2 and PD-L1 expression in a cohort of 437 oesophageal cancers

**DOI:** 10.1038/s41416-020-0811-0

**Published:** 2020-03-25

**Authors:** Kazuo Okadome, Yoshifumi Baba, Daichi Nomoto, Taisuke Yagi, Rebecca Kalikawe, Kazuto Harada, Yukiharu Hiyoshi, Yohei Nagai, Takatsugu Ishimoto, Masaaki Iwatsuki, Shiro Iwagami, Yuji Miyamoto, Naoya Yoshida, Masayuki Watanabe, Yoshihiro Komohara, Takashi Shono, Yutaka Sasaki, Hideo Baba

**Affiliations:** 1 Department of Gastroenterological Surgery, Graduate School of Medical Sciences, Kumamoto University, 1-1-1 Honjo, Chuo-ku, Kumamoto, 860-8556 Japan; 2 Department of Next-Generation Surgical Therapy Development, Graduate School of Medical Sciences, Kumamoto University, 1-1-1 Honjo, Chuo-ku, Kumamoto, 860-8556 Japan; 3 Department of Gastroenterological Surgery, Cancer Institute Hospital, Japanese Foundation for Cancer Research, 3-8-31 Ariake, Koto-ku, Tokyo, 135-8550 Japan; 4 Department of Cell Pathology, Graduate School of Medical Sciences, Faculty of Life Sciences, Kumamoto University, 1-1-1 Honjo, Chuo-ku, Kumamoto, 860-8556 Japan; 5 Department of Gastroenterology and Hepatology, Graduate School of Medical Sciences, Kumamoto University, 1-1-1 Honjo, Chuo-ku, Kumamoto, 860-8556 Japan

**Keywords:** Cancer immunotherapy, Immunoediting

## Abstract

**Background:**

The PD-1/PD-L1 pathway plays critical roles in tumour immunology, and serves as an immune-based therapeutic target. Less is known regarding PD-L2, another ligand of PD-1, and its relation to clinical outcome in human cancers.

**Methods:**

We used a database of 437 surgically and 100 endoscopically resected oesophageal cancers (squamous cell carcinoma, *n* = 483; adenocarcinoma, *n* = 36; others, *n* = 18) to evaluate PD-L2 and PD-L1 expression by immunohistochemistry.

**Results:**

Compared with PD-L2-negative cases (*n* = 366, 83.8%), PD-L2-positive cases (*n* = 71, 16.2%) had worse overall survival (*P* = 0.011, log-rank test). There was not a significant correlation between PD-L2 and PD-L1 expression. Multiplex immunofluorescence revealed that there was variability in the expression pattern of PD-L2 and PD-L1. In early-stage tumours, PD-L2 expression was more frequently observed compared with PD-L1.

**Conclusions:**

PD-L2 as well as PD-L1 were associated with an unfavourable prognosis in oesophageal cancer, supporting the role of PD-L2 as a prognostic biomarker. Considering that PD-L2 and PD-L1 had different features in terms of expression timing and responses to chemotherapeutic drugs, evaluation of both PD-L2 and PD-L1 expression may be clinically important.

## Background

Oesophageal cancer is the sixth leading cause of cancer-related death and the seventh most common cancer worldwide.^1^ Despite the development of multimodal therapies, including surgery, chemotherapy and chemoradiotherapy, the prognosis of oesophageal cancer patients, including those who undergo complete resection, remains poor because of the extremely aggressive nature of this cancer type and the poor survival rate.^2,3^ The limited improvement in treatment outcome by conventional therapies has prompted the search for innovative strategies for the treatment of oesophageal cancer, especially immunotherapeutically targeted treatments.^4,5^

The programmed death 1 (PD-1) pathway serves as a checkpoint to limit the T-cell-mediated immune responses. Two ligands, programmed death ligand 1 (PD-L1) and programmed death ligand 2 (PD-L2), engage the PD-1 receptor and induce PD-1 signalling and the associated T-cell exhaustion, a reversible inhibition of T-cell activation and proliferation. Tumour cells can co-opt the PD-1 pathway to evade the immune response by expressing PD-1 ligands on the cell surface and engaging PD-1 receptor-positive immune effector cells.^6–8^ Recently, many clinical trials have demonstrated that PD-1/PD-L1 signal-blockade agents show dramatic antitumour efficacy in patients with many types of malignancies, including oesophageal cancer.^9–13^ PD-L1 expression is not only a predictive biomarker for the response to immunotherapy,^14,15^ but it is also a prognostic factor for several types of cancers.^16,17^ However, PD-L2, another PD-1 ligand, has gained little attention, and its clinical and prognostic characteristics in oesophageal cancer remain unclear.^18–20^ Given that PD-L2 demonstrates higher affinity with PD-1 compared with PD-L1,^21,22^ we hypothesised that PD-L2 expression might indicate oesophageal cancer with aggressive biological behaviour. Considering the increasing clinical importance of immune checkpoint inhibitors, the assessment of PD-L2 and PD-L1 expression and clinical outcome using a large number of oesophageal cancers is needed.

To test our hypothesis, we evaluated PD-L2 as well as PD-L1 expression by immunohistochemistry in a large cohort of 437 oesophageal cancer patients, and examined the prognostic impacts in oesophageal cancer. To the best of our knowledge, this is the first study to assess the prognostic features of the combination of PD-L2 and PD-L1 status in more than 400 patients with surgically resected oesophageal cancer.

## Methods

### Patients

A total of 495 consecutive patients with oesophageal cancer who were undergoing curative resection at Kumamoto University Hospital between April 2005 and March 2016 were enroled in this study. Thirty-three patients who did not have assessable cancer cells were excluded, as well as 25 patients without clinical data. A total of 437 oesophageal cancer patients were finally included in this study. The most frequent histological type was squamous cell carcinoma (SCC, 383 patients, 87.6%), followed by adenocarcinoma (*n* = 36, 8.2%) and others (*n* = 18, 4.1%). There were 159 cases with preoperative chemotherapy or chemoradiotherapy. Most patients had received fluorouracil/cisplatin/docetaxel (*n* = 111, 69.8%) or fluorouracil/cisplatin (*n* = 39, 24.5%). Four cases had received other chemotherapeutic agents. In five cases, the regimen was unknown. Tumour stage was classified according to the eighth edition of the American Joint Committee on Cancer TNM classification system.^23^ Patients were followed up as outpatients every 1–3 months after discharge until death or December 2018. Overall survival was defined as the period from the date of surgery to the date of death. To evaluate the PD-L1 and PD-L2 expression status in early-stage cancer, we enroled 100 patients with oesophageal squamous cell carcinoma, who underwent endoscopic submucosal dissection (ESD) at Kumamoto University Hospital. Among the ESD cases, 23 cases showed tumour depth of pT1a epithelium (EP) (= Tis), 22 cases were pT1a-lamina propria (LPM), 25 cases were pT1a-muscularis mucosae (MM) and 30 cases were pT1b submucosa (SM). No patient received chemotherapy or chemoradiotherapy before ESD treatment. Written informed consent was obtained from each subject, and the study procedures were approved by the Institutional Review Board of Kumamoto University. Throughout this paper, the term ‘prognostic marker’ is used according to REMARK Guidelines.^24^

### PD-L1 and PD-L2 immunohistochemical staining

For PD-L2 staining, tumour sections (4 µm) were dewaxed and rehydrated, and antigen retrieval was performed by adding pH9 antigen retrieval solution (Histofine; Nichirei, Tokyo, Japan) and autoclaving at 121 °C for 15 min. After neutralisation of endogenous peroxidase, the sections were pre-incubated with blocking serum and incubated overnight at 4 °C with a primary antibody against PD-L2 (1:200, clone D7U8C, Cell Signaling Technology, MA, USA). The sections were incubated with biotinylated secondary antibodies and avidin–biotin complex method reagent (Vectastain Elite ABC Kit, Vector Laboratories, Burlingame, CA, USA). Mayer’s haematoxylin was used for counterstaining. We scored PD-L2 expression in tumour cells according to expression level (no expression = score 0, weak expression = score 1, moderate expression = score 2 or strong expression = score 3) (Supplementary Fig. 1a) and proportion of stained tumour cells (0–10%=score 0, 10–30% = score 1, 30–50% = score 2 or over 50% = score 3). The total score was calculated as the product of the expression and proportion score, and PD-L2-positive expression was determined as a score of 3 or more. PD-L1 staining and evaluation of PD-L1 expression were conducted as previously described (Supplementary Fig. 1b).^17^ PD-L1- and PD-L2-stained tissue sections were reviewed by two pathologists (Y.B. and Y.K.) who were unaware of other data.

### Double immunohistochemical staining

Both PD-L1- and PD-L2-positive cases were subjected to double immunohistochemical staining. Briefly, immunohistochemical staining of PD-L2 and visualisation with DAB (DACO, Carpinteria, CA, USA) was performed as described above. After heating in a microwave at 600 W for 3 min, PD-L1 staining was then performed and visualised by HistoGreen (Cosmo Bio, Tokyo, Japan).

### Multiplex immunofluorescence

For multiplex immunohistochemical staining, anti-PD-L1, PD-L2 antibody described above and Opal 4-Color fluorescent IHC kit (PerkinElmer, Waltham, MA, USA) were used. PD-L1 was optimised using Opal 470 Fluorophore (red), and then PD-L2 was optimised using Opal 520 Fluorophore (green) according to the manual of the Opal IHC kit. Finally, VECTASHIELD mounting medium with DAPI (Vector) was used to stain nuclei.

### Tumour-infiltrating lymphocyte evaluation

H&E-stained tissue sections were reviewed to assess tumour-infiltrating lymphocytes by a pathologist (Y.B.) who was unaware of other data. Lymphocyte infiltration in the tumour- invasive margin was scored as absent, mild, moderate or strong.

### Quantitative real-time reverse transcription–polymerase chain reaction

Twenty-five pairs of primary oesophageal squamous cell carcinoma tissues and matched oesophageal epithelia were obtained from resected oesophageal cancer patients to evaluate *PD-L2* expression. Total RNA was isolated from tissue samples using an RNeasy Mini Kit (Qiagen, Hilden, Germany), according to the manufacturer’s protocol. The mRNA expression levels were determined by quantitative real-time reverse transcription–polymerase chain reaction (qRT–PCR) using TaqMan probes (Roche, Basel, Switzerland), and were normalised to those of β-actin. To design the qRT–PCR primers, we accessed the Universal Probe Library (Genenet, Fukuoka, Japan), following the manufacturer’s recommendations. Real-time PCR was performed with the following primer sequences and probes: PD-L2 (PD-L2_#36), 5′-AAAGAGGGAAGTGAACAGTGC T-3′ and 5′-GCTTCTTTAGATGTCATATCAGGTCA-3′, and β-actin (ACTB_#11), 5′-ATTGGCAATGAGCGGTTC-3′ and 5′-CGTGGATGCCACAGGACT-3′. All qRT–PCR reactions were performed in the LightCycler 480 System II (Roche). All data for qRT–PCR were obtained from triplicate experiments, and are presented as the mean ± standard error.

### Statistical analysis

All statistical calculations were performed with JMP version 13 software (SAS Institute, Cary, NC, USA). All *P* values were two-sided. Categorical variables were presented as numbers and percentages, and groups were compared using the *χ*^2^ test or Fisher’s exact test. Continuous variables were expressed as means and standard deviations, and means were compared using the t test. The survival time distribution was evaluated by the Kaplan–Meier method, and the log-rank test was used for comparisons. Variables for which the *P* value in the univariate analysis was <0.05 were subjected to multivariate analysis by a stepwise backward elimination procedure using a threshold *P* value of <0.05. *P* < 0.05 was considered statistically significant.

## Results

### PD-L2 expression in oesophageal cancer patients

**Fig. 1:**
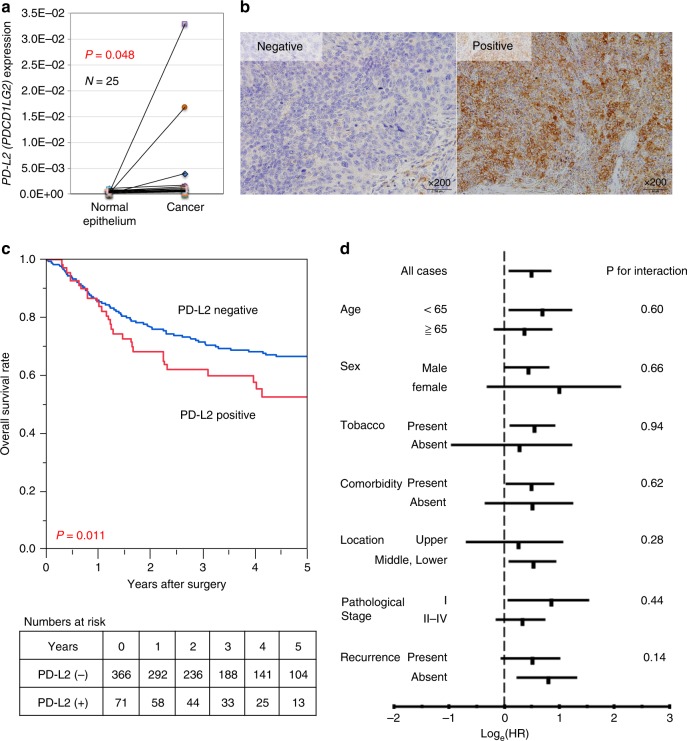
PD-L2 expression in oesophageal cancer and association with patient survival. **a** qRT–PCR analysis of *PD-L2* mRNA expression in 25 oesophageal cancer samples of matched normal epithelium and cancer. High *PD-L2* expression was observed in the tumour part compared with normal epithelium (*P* = 0.048). **b** Immunohistochemistry of PD-L2 expression in oesophageal cancer tumour sections. In PD-L2-positive cases, PD-L2 was stained in the cytoplasm and/or membrane of cancer cells. Original magnification, ×200. **c** Kaplan–Meier curves for overall survival in oesophageal cancer patients according to PD-L2 expression status. The PD-L2-positive group showed a significantly shorter overall survival than the PD-L2-negative group (log-rank *P* = 0.011). **d** Relationship between PD-L2 and overall survival. Log_e_ (hazard ratio) plots of overall survival rate in PD-L2-positive and PD-L2-negative groups are shown. The effect of PD-L2 was not significantly modified by age, sex, tobacco, comorbidity, location, pathological stage or recurrence (*P* > 0.14 for all interactions).

We evaluated the expression level of *PD-L2* mRNA by qRT–PCR in 25 oesophageal cancer tissues, and matched normal oesophageal epithelium. *PD-L2* expression in oesophageal cancer was significantly higher than that in normal oesophageal epithelium (*P* = 0.048) (Fig. 1a). In the PD-L2-positive cases confirmed by immunohistochemical staining, PD-L2 expression was elevated in cancer cells compared with normal oesophageal epithelium (Supplementary Fig. 1c). Tumour samples from the 437 oesophageal cancer cases included in this study were examined for PD-L2 expression by immunohistochemistry, and scored as described in ‘Methods’. Among the 437 cases, 71 (16.2%) were in the PD-L2-positive group, and 366 (83.8%) were in the PD-L2-negative group. Representative immunohistochemical staining is shown in Fig. 1b.

### PD-L2 expression and patient survival

Among the 437 oesophageal cancer patients, 152 deaths occurred, including 95 oesophageal cancer-specific deaths, and 135 recurrences of oesophageal cancer were observed. The median follow-up time of censored patients was 4.4 years. Kaplan–Meier analysis indicated that the PD-L2-positive group had significantly shorter overall survival (*P* = 0.011, log-rank test) than the PD-L2-negative group (Fig. 1c). The 3-year overall survival rate was 62.1% in the PD-L2-positive group, and 71.5% in the PD-L2-negative group. In the univariate Cox regression analyses, patients in the PD-L2-positive group showed significantly higher overall mortality than those in the PD-L2-negative group [hazard ratio (HR): 1.63; 95% confidence interval (CI): 1.09–2.36; *P* = 0.018]. Adjusting for clinical, epidemiological and pathological features in the multivariate Cox model, PD-L2 was associated with significantly higher overall mortality (multivariate HR: 1.81; 95% CI: 1.21–2.63; *P* = 0.004) (Table 1). We also employed an additional multivariate Cox model including PD-L1, and found that PD-L2 expression was an independent prognostic factor (multivariate HR: 1.75; 95% CI: 1.17–2.55; *P* = 0.007). We next determined whether the influence of PD-L2 on overall survival was affected by clinical, pathological or epidemiological variables. The effect of PD-L2 was not significantly modified by age, sex, tobacco use, comorbidity, location, pathological stage or recurrence (*P* > 0.14 for all interactions) (Fig. 1d).

**Table 1:** Univariate and multivariate analysis of overall survival.

Variables	Univariate analysis		Multivariate analysis	
	HR (95% CI)	*P*	HR (95% CI)	*P*
Age (≥65 vs. <65)	1.38 (0.99–1.93)	0.097		
Sex (male vs. female)	1.37 (0.81–2.54)	0.61		
Tobacco use (yes vs. no)	0.97 (0.67–1.53)	0.12		
Body mass index (<22 vs. ≥22)	1.17 (0.85–1.63)	0.94		
Comorbidity (present vs. absent)	1.33 (0.92–1.98)	0.13		
Location (upper vs. middle and lower)	1.20 (0.78–1.80)	0.39		
Pathological stage (II–IV vs. I)	2.53 (1.77–3.71)	<0.001	1.48 (1.00–2.23)	0.048
Recurrence (present vs. absent)	4.88 (3.51–6.83)	<0.001	4.41 (3.10–6.33)	<0.001
Adjuvant therapy (present vs. absent)	1.17 (0.81–1.67)	0.39		
PD-L2 (positive vs. negative)	1.63 (1.09–2.36)	0.018	1.81 (1.21–2.63)	0.004

*HR* hazard ratio, *CI* confidence interval, *PD-L2* programmed death ligand 2.

### PD-L2 and PD-L1 expressions and clinicopathological features

**Fig. 2:**
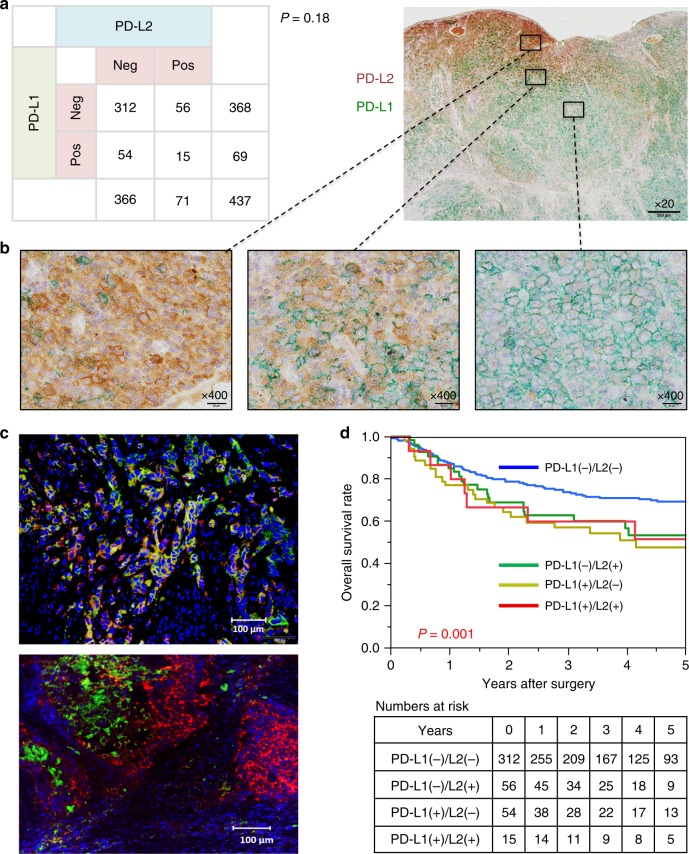
The relationship between PD-L1 and PD-L2 expression and association with patient survival. **a** The relationship between PD-L1 and PD-L2 expression status. There was no significant correlation between PD-L1 and PD-L2 expression (*P* = 0.18) (*n* = 437). **b** Double immunohistochemical staining of PD-L1 and PD-L2. Green areas show PD-L1 expression and brown areas show PD-L2 expression. (Left) Cancer cells expressing only PD-L2, (Middle) cancer cells expressing both PD-L1 and PD-L2 and (Right) cancer cells expressing only PD-L1. **c** Multiplex immunohistochemical staining of PD-L1 and PD-L2. FITC was used to visualise PD-L2 (green colour), Cy5 was used to visualise PD-L1 (red colour) and DAPI was used to visualise nuclei (blue colour). (Top) Cancer cells expressing PD-L2 and PD-L1 were mixed. (Bottom) PD-L2- and PD-L1-expressing cells have different location. **d** Kaplan–Meier curves for overall survival in oesophageal cancer patients according to PD-L1 and PD-L2 expression status. Both PD-L1- and PD-L2-negative group showed significantly favourable overall survival compared with the other groups (log-rank *P* = 0.001).

We next evaluated PD-L1 expression in the 437 cases and categorised the cases as described in Methods. Among the 437 cases, 69 (15.8%) were in the PD-L1-positive group, and 368 (84.2%) were in the PD-L1-negative group. Importantly, there was no significant correlation between PD-L1 and PD-L2 expression (*P* = 0.18) (Fig. 2a). Table 2 summarises the clinicopathological features of all cases. PD-L2 was significantly associated with age (*P* = 0.029), while PD-L1 was significantly associated with tobacco use (*P* = 0.013), receiving preoperative therapy (*P* = 0.015) and the pathological stage (*P* = 0.001). The chemotherapy regimen did not influence the relationship between PD-L1 expression and preoperative chemotherapy. Considering that the PD-L1/L2 status may influence the status of tumour-infiltrating lymphocytes, we examined the relationship between PD-L1/L2 and tumour-infiltrating lymphocyte statuses. As a result, the tumour-infiltrating lymphocyte status was significantly associated with PD-L1 expression (*p* = 0.030), but not PD-L2 expression (*p* = 0.42). Taken together, these results suggest that PD-L2- and PD-L1-positive tumours might present with different clinical features.

**Table 2:** Patient characteristics.

Variables	PD-L1			PD-L2		
	Negative *N* = 368	Positive *N* = 69	*P* value	Negative *N* = 366	Positive *N* = 71	*P* value
Age (y), mean ± SD	65.9 ± 9.0	67.4 ± 9.0	0.90	66.5 ± 9.0	64.3 ± 8.8	0.029
Sex			0.39			0.65
Male	328 (89.1)	59 (85.5)		323 (88.3)	64 (90.1)	
Female	40 (10.9)	10 (14.5)		43 (11.8)	7 (9.9)	
Body mass index, mean ± SD	21.8 ± 3.0	21.5 ± 3.3	0.50	21.7 ± 3.1	21.8 ± 3.1	0.72
Tobacco use			0.013			0.41
Yes	316 (85.9)	51 (73.9)		305 (83.3)	62 (87.3)	
No	52 (14.1)	18 (26.1)		61 (16.7)	9 (12.7)	
Alcohol use			0.10			0.46
Yes	325 (88.3)	56 (81.2)		321 (87.7)	60 (84.5)	
No	43 (11.7)	13 (18.8)		45 (12.3)	11 (15.5)	
Comorbidity			0.23			0.70
Present	262 (71.2)	54 (78.3)		266 (72.7)	50 (780.4)	
Absent	106 (28.8)	15 (21.7)		100 (27.3)	21 (29.6)	
Histological type			0.32			0.86
Squamous cell carcinoma	319 (86.7)	64 (92.8)		322 (88.0)	61 (85.9)	
Adenocarcinoma	32 (8.7)	4 (5.8)		29 (7.9)	7 (9.9)	
Others	17 (4.6)	1 (1.5)		15 (4.1)	3 (4.2)	
Location			0.64			0.29
Upper	59 (16.0)	8 (11.6)		54 (14.8)	13 (18.3)	
Middle	171 (46.5)	34 (49.3)		168 (45.9)	37 (52.1)	
Lower	138 (37.5)	27 (39.1)		144 (39.3)	21 (29.6)	
pStage			0.001			0.94
I	159 (43.2)	14 (20.3)		145 (39.6)	28 (39.4)	
II	97 (26.4)	19 (27.5)		99 (27.1)	17 (23.9)	
III	10 (27.2)	32 (46.4)		109 (29.8)	23 (32.4)	
IV	12 (3.3)	4 (5.8)		13 (3.6)	3 (4.2)	
Preoperative therapy			0.015			0.81
Present	125 (34.0)	34 (49.3)		134 (36.6)	25 (35.2)	
Absent	243 (66.0)	35 (50.7)		232 (63.4)	46 (64.8)	
Tumour-infiltrating lymphocytes (at the invasive margin)			0.030			0.42
Absent	8 (2.2)	0 (0)		7 (1.9)	1 (1.4)	
Mild	121 (32.9)	18 (26.1)		119 (32.5)	20 (28.2)	
Moderate	171 (46.5)	28 (40.6)		169 (46.2)	30 (42.3)	
Strong	68 (18.5)	23 (33.3)		71 (19.4)	20 (28.2)	

*PD-L1* programmed death ligand 1, *PD-L2* programmed death ligand 2.

### Double immunohistochemical staining for PD-L2 and PD-L1

To evaluate the localisation of PD-L2 and PD-L1 expression in oesophageal cancer tissues, we first performed double immunohistochemical staining. In most cases, heterogeneity of PD-L2 and PD-L1 expression in tumours was observed. Even in tumours with both PD-L2- and PD-L1-positive cases, cancer cells do not necessarily express both PD-L2 and PD-L1 (Fig. 2b); cancer cells with only PD-L2 expression and those with only PD-L1 expression exist simultaneously in one tumour. These findings were confirmed by using multiplex immunofluorescence assay (Fig. 2c). These results support the possibility that PD-L2 and PD-L1 might play different roles in tumour immunity in oesophageal cancers.

### PD-L1 and PD-L2 status and patient survival

We previously reported that PD-L1 expression was associated with unfavourable clinical outcome in oesophageal cancer.^17^ Given that the assessment of both PD-L2 and PD-L1 expression and clinical outcome may be clinically important, we classified the oesophageal cancer cases into four groups based on PD-L2 and PD-L1 expression. Kaplan–Meier analysis showed that the group negative for both PD-L1 and PD-L2 experienced significantly favourable overall survival (log-rank *P* = 0.001) compared with the other groups, either single or double positive for PD-L1 and PD-L2 (Fig. 2d). In the univariate Cox regression analyses, patients negative for both PD-L1 and PD-L2 showed significantly reduced overall mortality compared with groups positive for either ligand or both (HR: 0.52; 95% CI: 0.37–0.72; *P* < 0.001). In the multivariate Cox model, the group negative for both PD-L1 and PD-L2 was associated with significantly reduced overall mortality (multivariate HR: 0.56; 95% CI: 0.40–0.79; *P* < 0.001). We examined the clinical characteristics of the group negative for both PD-L1 and PD-L2. The PD-L1- and PD-L2-negative group had more non-advanced-stage patients than PD-L1- and/or PD-L2-positive groups, but there were no significant differences in other factors (Supplementary Table 1). In the correction for multiple comparison of factors associated with PD-L1/L2 status, pathological stage (multivariate OR: 0.59; 95% CI: 0.36–0.95; *P* = 0.030), and tumour-infiltrating lymphocytes (multivariate OR: 0.62; 95% CI: 0.38–0.99; *P* = 0.048) were significantly associated with both PD-L1- and PD-L2-negative groups. These findings indicate the need to evaluate not only PD-L1 but also PD-L2 expression in oesophageal cancers.

### Differences in timing of PD-L1 and PD-L2 expression during tumour development

**Fig. 3:**
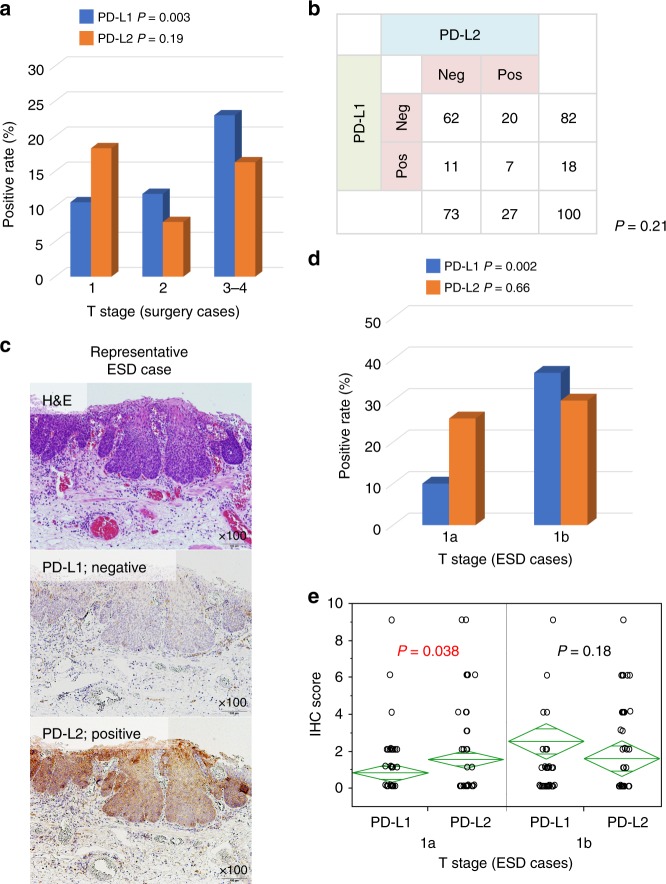
Examination of PD-L1 and PD-L2 expression in early-stage patients using ESD samples. **a** PD-L1 expression was significantly associated with tumour depth (*P* = 0.003), and no relationship was observed between PD-L2 expression and tumour depth (*P* = 0.19) in surgically resected samples. **b** The relationship between PD-L1 and PD-L2 expression status in ESD samples. There was no significant correlation between PD-L1 and PD-L2 expression (*P* = 0.21) (*n* = 100). **c** PD-L1-negative and PD-L2-positive expression in early-stage oesophageal cancer patients with ESD samples. Original magnification, ×100. **d** The positive rate of PD-L1 and PD-L2 for T1a and T1b tumour depth in 100 ESD samples. The positive rates of PD-L1 and PD-L2 were 10% and 25.7% for pT1a, and 36.7% and 30% for pT1b, respectively. **e** The relationship between immunohistochemistry (IHC) score of PD-L1/L2 and tumour depth. The IHC score was calculated as the product of the expression and proportion scores.

As previously indicated, PD-L1 expression was significantly associated with pathological stage (*P* = 0.001) and tumour depth (*P* = 0.013) (Fig. 3a), and frequently observed in advanced-stage tumours. In contrast, no relationship was observed between PD-L2 expression and pathological stage (*P* = 0.94); PD-L2 positivity was observed in both early- and advanced-stage tumours. Based on these findings, we hypothesised that there may be a difference in emergence of PD-L2 and PD-L1 expression during tumour development. To test this hypothesis, we examined the frequency of PD-L2 and PD-L1 positivity in 100 ESD cases with early-stage tumours because ESD is often performed for cases with superficial oesophageal cancer without metastasis in Japan.^25,26^ Out of 100 cases, 27 (27%) cases were PD-L2 positive and 18 (18%) cases were PD-L1 positive. In the ESD samples, there was no significant relationship between PD-L2 and PD-L1 expression (*P* = 0.21) (Fig. 3b). A representative case is shown in Fig. 3c. In early-stage cases, the frequency of PD-L1- positive cases increased as the tumour progressed in depth, but PD-L2 did not have a relation with the depth; dividing cases into pT1a and pT1b cases by depth revealed that the positive rates of PD-L1 and PD-L2 were 10% and 25.7% for pT1a, and 36.7% and 30% for pT1b, respectively (Fig. 3d). In comparing the immunohistochemistry score, the expression of PD-L2 was significantly higher than PD-L1 (*P* = 0.038), with an average of 0.86 for PD-L1 and 1.57 for PD-L2 in pT1a (Fig. 3e). There was no significant difference in scores among pT1b cases (2.57 and 1.63, respectively) (*P* = 0.18).

### The influence of preoperative therapy on PD-L1 and PD-L2 expression

Clinical data showed that PD-L1-positive cases were significantly associated with preoperative therapy-treated cases (*P* = 0.015), while PD-L2-positive cases did not show this association (*P* = 0.81). We further examined the positive rate of PD-L1 and PD-L2 in the no preoperative therapy group, preoperative chemotherapy group and preoperative chemoradiotherapy group (Supplementary Fig. 1d). The positive rate of PD-L1 was higher in patients who received preoperative chemotherapy (21.8%; *P* = 0.023) or chemoradiotherapy (20.4%; *P* = 0.143) than in patients who did not receive preoperative therapy (12.6%). The positive rate of PD-L2 was not significantly altered in these groups (16.6%, 17.3% and 12.2%, respectively). To clearly assess the effect of chemotherapy on PD-L2 expression, we examined pre-treatment status of PD-L2 expression using biopsy samples. However, the PD-L2 expression status in resected and biopsy specimens does not necessarily correlate (data not shown). This might be due to the heterogeneity of PD-L1/L2 expression in tumours.

## Discussion

In this study, we evaluated PD-L2 as well as PD-L1 expression by immunohistochemistry in more than 400 patients with surgically resected oesophageal cancer, and examined the prognostic impacts of PD-L1 and PD-L2 expressions in oesophageal cancer. PD-L2 and PD-L1 expression was associated with an unfavourable clinical outcome of oesophageal cancer, suggesting that the PD-L2 expression status may serve as a biomarker to identify patients who are likely to experience an unfavourable clinical outcome. In addition, this is the first study to assess the prognostic features of the combination of PD-L2 and PD-L1 statuses in more than 400 patients with surgically resected oesophageal cancer. We also found that PD-L2 and PD-L1 showed different expression timings during tumour development, and various responses to chemotherapeutic agents. Considering that immunotherapies, such as immune checkpoint inhibitors, have gained increasing attention as a novel treatment strategy for oesophageal cancer, our observations may have clinical implications.

The importance of PD-L1 expression as a predictive and prognostic biomarker in human cancers has been well studied. Nonetheless, PD-L2, another ligand of PD-1, has gained less attention. Whether PD-L2 expression correlates with prognosis in solid cancer patients after surgery remains elusive. With regard to oesophageal cancer patients, some papers reported that high PD-L2 expression in oesophageal cancer was associated with impaired survival,^18,19^ and another study showed no correlation between PD-L2 and clinical outcome.^20^ However, all of these studies were limited by small sample sizes (*n* ≤ 180) and low statistical power. In contrast to previous studies, our study evaluated PD-L2 expression by immunohistochemistry in a much larger cohort of oesophageal cancers.

Both PD-L2 and PD-L1 are B7 family members, and binding of these ligands to the receptor PD-1 results in inhibition of T- and B-cell responses.^6^ Although there are many similarities between PD-L2 and PD-L1, some differences have been reported in regard to localisation,^8^ receptors,^27^ affinity with their receptor PD-1^21,22^ and response to cytokines.^28^ The relationship between PD-L1 and PD-L2 expression in various types of cancers is still controversial.^29–32^ In this study, our results showed no significant relationship between PD-L1 and PD-L2 expression in oesophageal cancer. In addition, our multiple immunohistochemical staining and multiplex immunofluorescence showed the heterogeneity of PD-L1 and PD-L2 expression. Cancer cells with only PD-L2 expression and those with only PD-L1 expression exist simultaneously in tumours with both PD-L2- and PD-L1-positive cases. We thus speculate that PD-L2 might exhibit different functions than PD-L1 in oesophageal cancer development and progression.

Simultaneous assessment of not only PD-L1 but also PD-L2 for the therapeutic effect of immune checkpoint inhibitors may be clinically important. Yearley et al.^33^ reported that PD-L2 status was a significant predictor of progression-free survival with pembrolizumab independent of PD-L1 status, and the objective response rate was the highest in patients expressing both PD-L1 and PD-L2 in head and neck squamous cell carcinoma cases. Simultaneous evaluation of PD-L2 along with PD-L1 may result in an increased number of patients indicated for PD-1/PD-L1 signal-blockade agents; these drugs may yield positive results in patients negative for PD-L1 and positive for PD-L2. In this study, the assessment of the combination of PD-L2 and PD-L1 could enable further classification of patients according to clinical outcome. Our results revealed two differences between PD-L1 and PD-L2 expression in oesophageal cancers. One is the relationship between PD-L1 and PD-L2 expression and tumour stage. PD-L1 expression may increase along with tumour progression, while PD-L2 expression occurred relatively earlier compared with PD-L1. Another observation from our results is the relationship between PD-L1, PD-L2 and chemotherapy. Recently, the precise strategy for administering chemotherapy and immune checkpoint inhibitor in combination or sequentially has been debated.^34–36^ Our clinical data demonstrate that preoperative treatment is associated with PD-L1 expression, but not PD-L2 expression. Although PD-L1 expression is known to be upregulated due to various chemotherapeutic drugs or radiation therapies,^37–42^ only one report has examined the effect of these treatments on PD-L2^43^ (Supplementary Table 2). A better understanding of the influence of chemotherapeutic drugs on PD-L1 and PD-L2 expression may enable more accurate prediction of the treatment effect of immune checkpoint inhibitors.

In summary, our large cohort study, including more than 400 cases, revealed that PD-L2 expression, as well as PD-L1, was associated with an unfavourable clinical outcome in oesophageal cancer, supporting the role of PD-L2 as a prognostic biomarker. In addition, we found that PD-L2 and PD-L1 presented different features in terms of the expression timing and the response to chemotherapeutic drugs. Considering that drugs targeting the PD-1 pathway are being intensively developed and tested in clinical trials for various human cancers, PD-L1 and PD-L2 expression in oesophageal cancer may serve as a predictive biomarker, and may be used for patient selection in clinical trials of such drugs.

## Supplementary information

Supplemental figure and tables

## Data Availability

The datasets used and/or analysed during this study are available from the corresponding author on reasonable request.
