# Supplementary material for: Prognostic and clinical impact of PD-L2 and PD-L1 expression in a cohort of 437 oesophageal cancers
Source: Br J Cancer. 2020 Mar 25;122(10):1535–43. doi: 10.1038/s41416-020-0811-0 (PMC7217865; doi:10.1038/s41416-020-0811-0)
Supplement: Supplementary file 1 — Supplemental figure and tables [file 41416_2020_811_MOESM1_ESM.pdf]

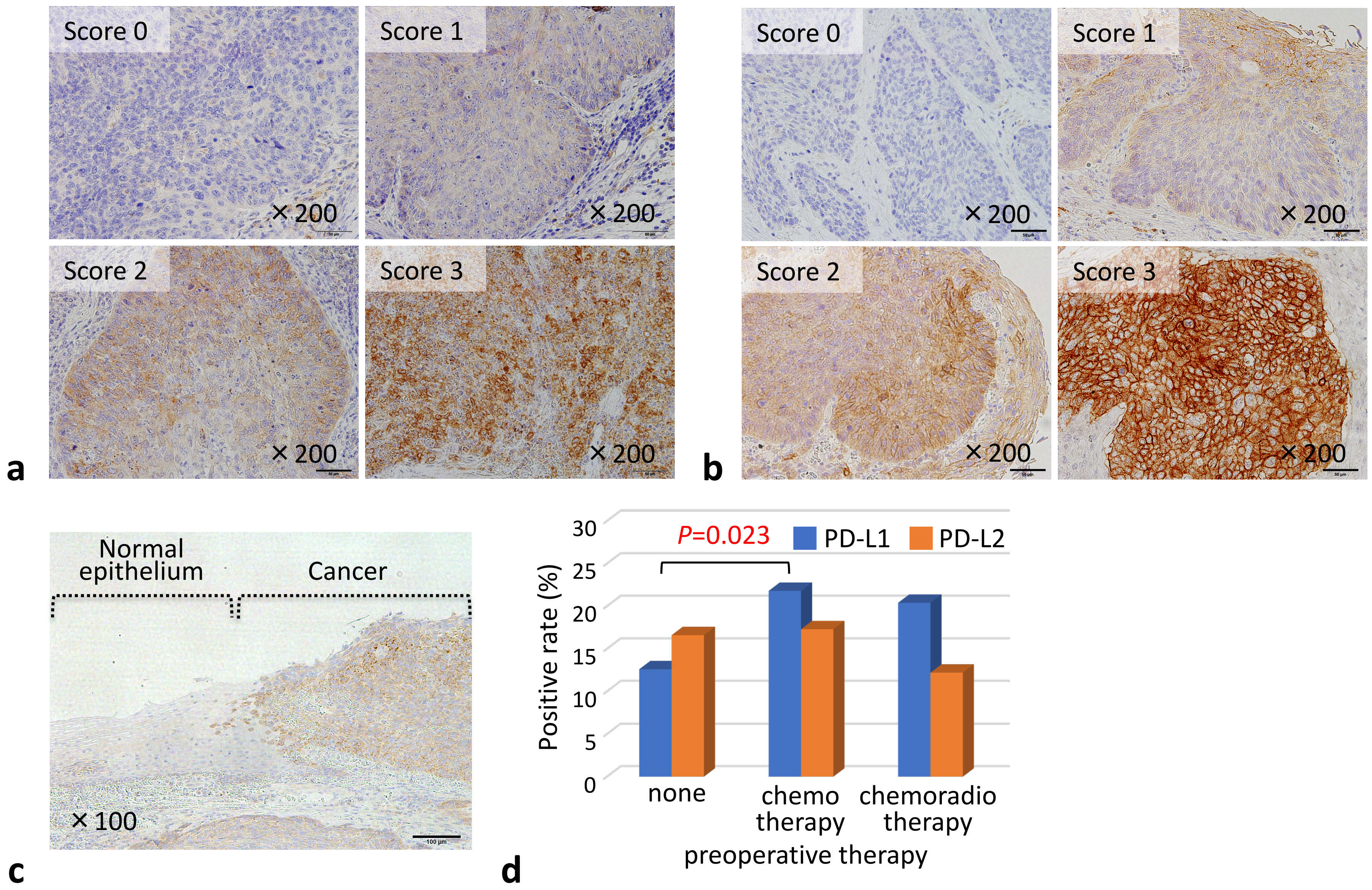

Supplemental figure 1

a. Intensity of IHC staining for PD-L2 expression of cancer cell was graded as absent (score 0), weak (score 1), moderate (score 2), or strong (score 3).
b. Intensity of IHC staining for PD-L1 expression of cancer cell was graded as absent (score 0), weak (score 1), moderate (score 2), or strong (score 3).
c. In PD-L2-positive cases, PD-L2 was stained in the cytoplasm and/or membrane of cancer cells and showed almost no staining in normal epithelium.
d. The positive rate of PD-L1 was higher in patients who received preoperative chemotherapy (21.8%; *P*=0.023) or chemoradiotherapy (20.4%; *P*=0.143) than in patients who did not receive preoperative therapy (12.6%). The positive rate of PD-L2 was not significantly different in the three groups (16.6%, 17.3%, and 12.2%, respectively).

Supplemental table 1. Patient Characteristics

Variables	PD-L1 and L2 status		*P* Value
	Both negative N=312	PD-L1 and/or PD-L2 positive N=125	
Age (y), mean±SD	66.2±9.0	66.1±9.1	0.95
Sex			0.87
Male	277 (88.8)	110 (88.0)	
Female	35 (11.2)	15 (12.0)	
Body mass index, mean±SD	21.8±3.1	21.6±3.1	0.58
Tobacco use			0.39
Yes	265 (84.9)	102 (81.6)	
No	47 (15.1)	23 (18.4)	
Alcohol use			0.35
Yes	275 (88.1)	106 (84.8)	
No	37 (11.9)	19 (15.2)	
Comorbidity			0.54
Present	223 (71.5)	93 (74.4)	
Absent	89 (28.5)	32 (25.6)	
Histological type			0.72
Squamous cell carcinoma	271 (86.9)	112 (89.6)	
Adenocarcinoma	27 (8.7)	9 (7.2)	
Others	14 (4.5)	4 (3.2)	
Location			0.47
Upper	48 (15.4)	19 (15.2)	
Middle	141 (45.2)	64 (51.2)	
Lower	123 (39.4)	42 (33.6)	
pStage			0.034
I	134 (43.0)	39 (31.2)	
II	85 (27.2)	31 (24.8)	
III	75 (24.0)	45 (36.0)	
IV	18 (5.8)	10 (8.0)	
Preoperative therapy			0.09
Present	106 (34.0)	53 (42.4)	
Absent	206 (66.0)	72 (57.6)	
Tumor-infiltrating lymphocytes (at the invasive margin)			0.09
Absent	7 (2.2)	1 (0.8)	
Mild	104 (33.3)	35 (28.0)	
Moderate	145 (46.5)	54 (43.2)	
Strong	56 (18.0)	35 (28.0)	

PD-L1, programmed death ligand 1; PD-L2, programmed death ligand 2

Supplemental table 2. Alteration of PD-L1 or PD-L2 expression by previous treatment

**Therapeutic agent**			**PD-L1/L2**	**Cancer type**	**Ref.**
Chemotherapy					
	Cytotoxic	Fluorouracil	PD-L1 +	CRC, EAC	(37)
		Cisplatin	PD-L1 +	HNSCC	(38)
			**PD-L2 -**	Melanoma	(43)
		Paclitaxel	PD-L1 +	Breast Cancer	(39)
		Etoposide	PD-L1 +	Breast Cancer	(39)
	Molecular target	Trastuzumab	PD-L1 +	Breast Cancer	(40)
		Sunitinib	PD-L1 +	RCC	(41)
		Bevacizumab	PD-L1 +	RCC	(41)
Radiotherapy			PD-L1 +	Melanoma, Glioblastoma	(42)

PD-L2, programmed death ligand 2; PD-L1, programmed death ligand 1; CRC, colorectal cancer; EAC, esophageal adenocarcinoma; HNSCC, head and neck squamous cell carcinoma; RCC, renal cell carcinoma; Ref., reference
